# Standardized Genetic Transformation Protocol for Chrysanthemum cv. ‘Jinba’ with TERMINAL FLOWER 1 Homolog *CmTFL1a*

**DOI:** 10.3390/genes11080860

**Published:** 2020-07-28

**Authors:** Saba Haider, Yaohui Gao, Yike Gao

**Affiliations:** 1 National Flower Engineering Research Centre, Beijing Key Laboratory of Ornamental Plants Germplasm Innovation and Molecular Breeding, College of Landscape Architecture of Beijing Forestry University, Beijing 100083, China; sabahaider@bjfu.edu.cn; 2 Architectural Institute, Inner Mongolia University of Science & Technology, Alding Street No.7, Kundulun District, Baotou 014010, China; yaohui_gao@163.com

**Keywords:** *Chrysanthemum morifolium*, *CmTFL1a*, meropenem, transformation efficiency, expression pattern, transgenic plants

## Abstract

Chrysanthemum (*Chrysanthemum* x *morifolium* Ramat.) cultivar Jinba is a distinctive short-day chrysanthemum that can be exploited as a model organism for studying the molecular mechanism of flowering. The commercial value of Jinba can be increased in global flower markets by developing its proper regeneration and genetic transformation system. By addressing typical problems associated with *Agrobacterium*-mediated transformation in chrysanthemum, that is, low transformation efficiency and high cultivar specificity, we designed an efficient, stable transformation system. Here, we identify the features that significantly affect the genetic transformation of Jinba and standardize its transformation protocol by using *CmTFL1a* as a transgene. The appropriate concentrations of various antibiotics (kanamycin, meropenem and carbenicillin) and growth regulators (6-BA, 2,4-D and NAA) for the genetic transformation were determined to check their effects on in vitro plant regeneration from leaf segments of Jinba; thus, the transformation protocol was standardized through *Agrobacterium tumefaciens* (EHA105). In addition, the presence of the transgene and its stable expression in *CmTFL1a* transgenic plants were confirmed by polymerase chain reaction (PCR) analysis. The *CmTFL1a* transgene constitutively expressed in the transgenic plants was highly expressed in shoot apices as compared to stem and leaves. Overexpression of *CmTFL1a* led to a delay in transition to the reproductive phase and significantly affected plant morphology. This study will help to understand the biological phenomenon of *TFL1* homolog in chrysanthemum. Moreover, our findings can explore innovative possibilities for genetic engineering and breeding of other chrysanthemum cultivars.

## 1. Introduction

Chrysanthemum (*Chrysanthemum* x *morifolium* Ramat) has the largest floriculture commodity in China and is a popular traditional flower of esthetic and cultural importance. There is a growing demand for high quality chrysanthemum flowers and cut flowers in the local market. The plants can be regenerated from adventitious shoots from various chrysanthemum tissues or calli using in vitro culture methods [1]. There are various cultivars of chrysanthemum being commercially propagated, but Jinba holds significance due to its fast-growing habit. Jinba is a short-day chrysanthemum cultivar with relatively low transformation efficiency compared with other cultivars, making it difficult to obtain transgenic plants.

Efficient genetic transformation with the production of various transgenic crop plants is dependent on several factors, such as gene transfer methods, DNA recombinant technologies and tissue culture techniques [2,3,4]. Diverse methods for gene transfer have been developed, including *Agrobacterium*, particle bombardment, microinjection, in planta transformation and liposome encapsulation [5]. Among them, *Agrobacterium*-mediated transformation has a unique significance and is being used for the insertion of specific genes into numerous ornamental plant species because of its authenticity and reliability. Several studies have been reported concerning the evaluation of *Agrobacterium*-mediated transformation in chrysanthemum [6,7,8]. The susceptibility of chrysanthemum to *Agrobacterium*-mediated transformation is well established and has been characterized by low transformation efficiency since 1975 [9,10]. The formation of crown gall in various parts of *Chrysanthemum morifolium* due to B6 *Agro-*infection has been reported to be the main reason behind this low transformation efficiency. It was reported that regeneration of chrysanthemum is dependent on genotype, similar to tomato [11] and cucumber [12].

The most important feature that hinders the rapid advancement of genetically modified chrysanthemums is protocol reliance [13]. Each cultivar has a unique specificity for tissue culture and genetic manipulation methods. Therefore, no one protocol can be implemented for a wide range of cultivars. For Jinba, the genetic transformation has usually been performed with *A. tumefaciens*. Various factors significantly affect the rate of *Agrobacterium*-mediated transformation in chrysanthemum including the plant cultivar, source and size of explant, type of bacterial strain used, kind and concentration of selective agent used for the selection of genetically modified plants, time period and temperature of cocultivation and timing of *Agrobacterium* treatment [14]. A comprehensive evaluation of these factors is necessary to standardize the genetic transformation protocol for the Jinba cultivar of chrysanthemum plants.

Flowering is the major developmental phase, comprising of transition from vegetative to reproductive growth stages [15]. A comprehensive research on *TFL1* homologs has revealed diverse functions such as constitutive expression of *CsTFL*1 in chrysanthemum that caused delay in flowering time. Similar results were found in the ecotropic expression of maize *TFL1* homologs [16]. Several pathways regulate floral induction and flowering time to optimize adaptation and reproductive success in higher plants, including vernalization, plant hormone, photoperiodic and autonomous pathways. Thus, different plant species have developed their optimal approaches for the regulation of flowering time with environmental cues [15]. Two significant phosphatidylethanolamine-binding protein (PEBP) genes controlling these pathways are *FT* (*FLOWERING LOCUS T*) and *TFL1* (*TERMINAL FLOWER 1*) [17,18,19]. *TFL1* plays a key role in suppressing flowering by antagonizing the function of the *FT* protein, which is responsible for the promotion of flowering [20]. Moreover, it helps in the formation of inflorescences, maintains vegetative growth and an indeterminate inflorescence [21,22]. Several genes homologous to *TFL1* have been identified in many plants. Some of the examples include *Malus domestica* [23], *Chrysanthemum morifolium* [21] and *Chrysanthemum Seticuspe f. boreale* [24].

This work aims to enhance the number of putative regenerated shoots of Jinba by optimizing the protocol of *Agrobacterium*-mediated transformation, to maximize the feasibility of producing transgenic plants by standardizing media composition, and to examine the effect of *CmTFL1a* expression on the flowering of chrysanthemum plants by phenotypic analysis. *CmTFL1a was* isolated from *C. morifolium* Jinbudiao in our lab and its functional role in chrysanthemum was analyzed as mentioned in [21]. *CmTFL1a* was inserted into in vitro Jinba plants as a transgene to obtain transgenic lines. Various parameters responsible for successful transformation were assessed, including cocultivation period and cocultivation temperature at different intervals, for comprehensive screening. To ensure that the hormones and antibiotics used could enhance the regeneration capability of explants, various hormone and selective agent treatments at different concentrations were recorded. These include 6-benzylaminopurine (6-BA), naphthaleneacetic acid (NAA), 2,4-dichlorophenoxyacetic acid (2,4-D), carbenicillin, meropenem and kanamycin. The regenerated transgenic shoots were kept in rooting medium for one month then transferred into soil media. As a result, the maximum possibility of producing transgenic plants by standardizing the composition of media was achieved. Through repeated experiments, the combination of 6-BA and 2,4-D was proven to be most appropriate for producing a greater number of transgenic shoots. At the same time, the removal of agrobacterial strain was effectively accomplished by using meropenem, as compared to carbenicillin, at a specific concentration. Phenotypic analysis showed that *CmTFL1a*, like various *CEN/TFL1* genes, promotes vegetative growth, and negatively regulates the reproductive phase. It was found that overexpression of *CmTFL1a* significantly extended vegetative growth, delayed flowering and promoted atypical inflorescences in transgenic chrysanthemum plants.

## 2. Materials and Methods

### 2.1. Plant Material

A cultivar of *C. morifolium* named Jinba was grown in the greenhouse of Beijing Forestry University (Beijing, China). The optimal conditions for the photoperiod were maintained along with temperatures ranging between 18 °C and 25 °C.

#### 2.1.1. Preparation of Surface Sterilization Solution

In order to prepare 4% calcium hypochlorite (Ca(OCl)_2_), 10 g of (Ca(OCl)_2_) was dissolved in 250 mL of autoclaved distilled water and was stored at room temperature.

#### 2.1.2. Preparation of MS Media

For preparing 1 L of medium, the desired quantity of MS (for shoot initiation and multiplication media) and sucrose was dissolved in 300 mL distilled water following the recipe mentioned in Section 2.1.3. The required quantity of growth regulators was added in the medium after thawing followed by the addition of 400 mL of hot distilled water. After constant stirring, the pH was adjusted to 5.7. In another beaker, agar was added in 300 mL distilled water and was heated until the agar melted. The two solutions were mixed and stirred well, followed by pouring of medium in test tubes. Sterilization of the test tubes was performed by autoclaving for 15 min at 121 °C.

#### 2.1.3. In Vitro Propagation of Chrysanthemum Plants

In vitro propagation of Chrysanthemum was performed by taking pedicels as chrysanthemum explants from greenhouse plants. Pedicels with a diameter of 2–2.5 mm were harvested at the preflowering stage. The bottom part of the cuttings was removed and only pedicels (3 cm long) were used without any leaves. They were rinsed in tap water with 2 drops of colorless liquid soap for 3 min and then dried using filter paper. Surface sterilization was performed inside sterile conditions of a laminar flow hood by soaking the pedicels in a 4% calcium hypochlorite (Ca(OCl)_2_) for 15 min. Furthermore, the explants were rinsed with sterile distilled water for 2 min followed by changing the water at least three times. Fragments from the treated explants were prepared by cutting the pedicel into pieces 8–10 mm long. The semi-cylindrical fragments (one per tube) were incubated on 15 mL medium for shoot initiation (MS 4.4 g L^−1^ + Myoinositol 100 mg L^−1^ + 2 mg L^−1^ BAP (1 mL stock solution + IAA (1 mL stock solution) 1 mg L^−1^ + Sucrose 20 g/L + Agar 8 g L^−1^) at pH 5.7, and were cultivated at 24 °C, 80 µE m^−2^ s^−1^ and 16:8 h day-night photoperiod. Micropropagation was performed by gently cutting the shoots into microcuttings with one node and were incubated on 10 mL medium for multiplication (MS 4.4 g L^−1^ + Sucrose 20 g L^−1^ + Agar 6.5 g L^−1^) at pH 5.7 Jain and Ochatt [25].

### 2.2. Plant Expression Vector Construction

*Agrobacterium tumefacians* strain EHA105 carrying the binary vector pCAMBIA1301 with *CsTFL1a* was isolated from *C. morifolium* Jinbudiao under the control of cauliflower mosaic virus (CaMV) 35s promotor used for transformation. The full length *CmTFL1a* cDNA was obtained by PCR using primers *CmTFL1a*-F and *CmTFL1a*-R, whereas the products were purified using a PCR purification kit (AXGEN, USA). Cloning of amplified fragments into the pEASY-Blunt vector (TRANSGEN, Beijing, China) was accomplished and verified by sequencing. A pCAMBIA 1301– *pmi*- *CmTFL1a* overexpression vector was constructed with the Seamless Assembly Cloning Kit (CloneSmarter, Houston, TX, USA). An *Escherichia coli phosphomannose isomerase (PMI)* marker gene and mannose selective agent were used in a mannose selection system for transformation in chrysanthemum. Finally, amplification of *CmTFL1a* from pEASY-*CmTFL1a* with *CmTFL1a-pmi*-F and *CmTFL1a-pmi*-R primers was accomplished, followed by digestion of pCAMBIA 1301- *pmi* vector with *Xho*I, leading towards assembling of *CmTFL1a* into the plasmid using Seamless Assembly [21]. The experiment was repeated three times to ensure the best possible results.

### 2.3. Shoot Regeneration Medium

A total of 18 shoot regeneration media with various concentrations of hormones such as 6-BA, 2,4-D and NAA, and antibiotics such as carbenicillin, meropenem and kanamycin were prepared. Three distinct concentrations, which are listed in Table 1, were used for each hormone and antibiotic. The concentrations of the growth regulators 6-BA (0.5 mg L^−1^), 2,4-D (0.2 mg L^−1^) and NAA (0.1 mg L^−1^) [26]; and those of antibiotics carbenicillin (200 mg L^−1^) [13], meropenem (50 mg L^−1^) [27] and kanamycin (7 mg L^−1^) [28] were modified and used as controls. The purpose was to differentiate the impact of these hormones and antibiotics on the regeneration capability of treated leaves. Three biological replicates were used for each treatment by repeating the experiment thrice.

### 2.4. Agrobacterium-Mediated Transformation in Chrysanthemum

Chrysanthemum plants were genetically transformed via *A.tumefaciens* by following the unstandardized procedures of Naing et al. [29] with modifications. Leaf explants of Jinba were divided into small segments of 0.5–1 cm^2^ and dipped into *Agrobacterium* suspensions with various optical densities to determine the optimal optical density of *Agrobacterium*, which was directly proportional to the transformation efficiency. The time for suspension was optimized to 10 min. After suspension, the leaves were dried by blotting with filter paper before being placed on the shoot regeneration medium (MS + 0.5 mg L^−1^ 6BA + 0.1 mg L^−1^ NAA as control). Treated explants plated on cocultivation medium (MS + 0.5 mg L^−1^ 6BA + 0.1 mg L^−1^ NAA as control) with carbenicillin (400 mg L^−1^) were left for 2 days under dark conditions. Then, the leaves were transferred to selection medium (MS + 6-BA + NAA + Meropenem + Kanamycin) containing different concentrations of antibiotics. Stock solutions of these antibiotics were prepared by filter-sterilization at concentrations of 400 mg L^−1^, 50 mg L^−1^ and 30 mg L^−1^, respectively. Carbenicillin and meropenem ensure the removal of any residual bacteria, while kanamycin is a selective agent that promotes callus regeneration and shoot proliferation strength of the callus generated. Assessment of the factors affecting transformation efficiency was performed by manipulating different concentrations and combinations of growth regulators and antibiotics (Table 1), days to coculture and the temperature requirements during cocultivation. Each treatment contained 15 explants with 3 replications, whereas the experiment was conducted thrice.

### 2.5. PCR Analysis of Transgenic Plants

Genomic DNA was isolated from the leaves of transgenic and wild-type (WT) plants using a TIANGEN DNA secure Plant Kit followed by PCR analysis using specific primers and PCR parameters, as listed in Table 2. Analysis of PCR products was accomplished by gel electrophoresis using 2% agarose stained with ethidium bromide. The expression of the inserted transgene was analyzed by quantitative real-time PCR (qRT-PCR). Specific primers for qRT-PCR (Table 2) were designed by the Primer 5.0 software. The total volume of 20 µL mixture was prepared with 2 µL cDNA template (10X), 0.4 µL of each amplification primer (10 mmol L^−1^) and 10 µL 2x SYBR Green Master Mix (TAKARA, Japan). In all cases, the standard thermal cyclic conditions were: 50 °C for 2 min, 35 cycles of 95 °C for 10 min, 95 °C for 15 s, 60 °C for 15 s and 72 °C for 10 min. Three biological replicates were analyzed.

All the cultures were placed in a completely randomized design. A total of 18 treatments were used with three biological replicates. Each replicate dish had 5 explants; i.e., a total of 270 explants was used for genetic transformation. All the experiments were repeated three times. The data were analyzed using analysis of variance (ANOVA), and means were compared using Duncan’s multiple range test (*p* < 0.05).

## 3. Results

### 3.1. Role of the Cocultivation Period in Genetic Transformation Efficiency

**Figure 1:**
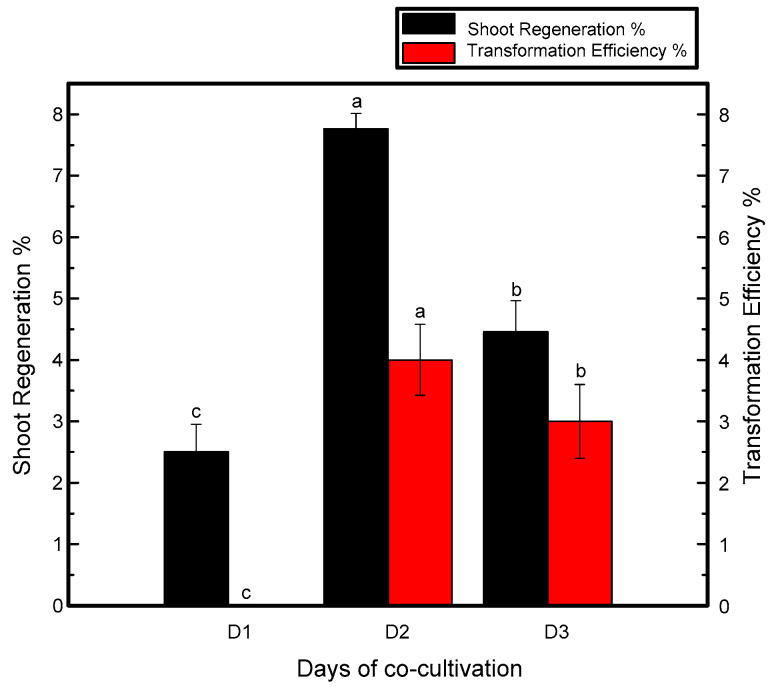
Relation of cocultivation period with shoot regeneration and transformation efficiency of Jinba. Black columns represent shoot regeneration percentage, red columns represent transformation efficiency percentage. Explants were cocultured for 1, 2 and 3 days. Three biological replicates were used. Error bars represent ± SD. a, b, and c are statistically significant at *p* ≤ 0.05 by ANOVA on ranks test.

Time of cocultivation may vary with the plant and species. The average time of cocultivation for chrysanthemum plants is two or four days [30,31]. Resistant shoots were not produced when the treated explants were cocultured for one day. However, the maximum number of putative shoots was observed when the explants were cocultured for two days in darkness, while the number of resistant shoots in the explants cocultured for more than two days was reduced, as shown in Figure 1.

### 3.2. Relation between cocultivation temperature and transformation efficiency

**Figure 2:**
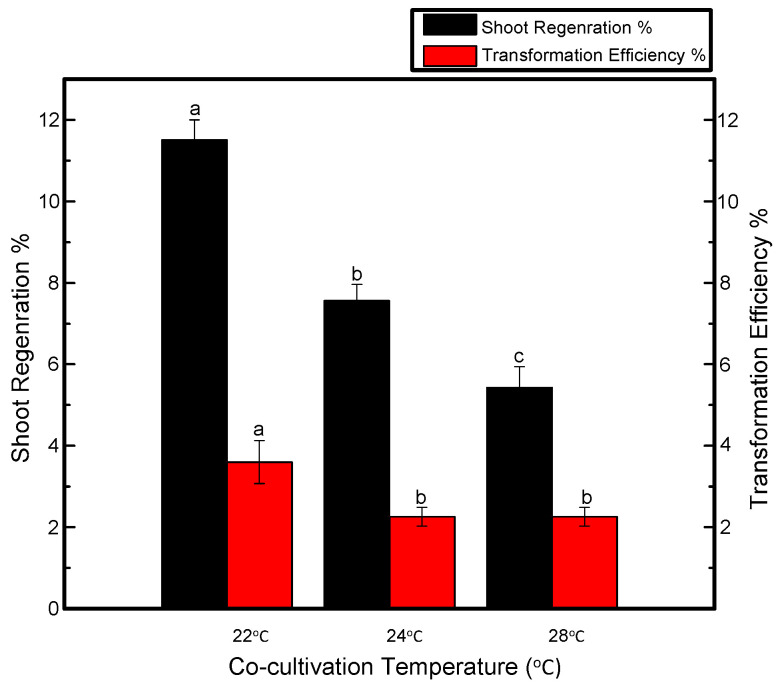
Effects of cocultivation temperature on shoot regeneration and transformation efficiency of Jinba. Black columns represent shoot regeneration percentage, red columns represent transformation efficiency percentage. Coculture plates were maintained at 22, 24 and 26 °C. Three biological replicates were used. Error bars represent ± SD. a, b, and c are statistically significant at *p* ≤ 0.05 by ANOVA on ranks test.

It can be seen in Figure 2 that temperature adjustment during cocultivation plays a crucial role in shoot regeneration. Explants were cocultivated at various temperature conditions and the maximum number of putative shoots was observed when the temperature maintained during the cocultivation period was 22 °C. However, explants cocultured at 24 °C yielded fewer transgenic shoots than those produced at 22 °C but more transgenic shoots than those produced at 28 °C. In fact, overgrowth of bacteria was observed after explants were transferred to the selection medium.

### 3.3. Role of Various Growth Regulators and Antibiotics on Shoot Regeneration

Minimal quantities of growth regulators and antibiotics that can promote shoot regeneration and help to destroy nontransgenic cells were investigated in the selection of transgenic plants. The growth regulators used included 6-benzylaminopurine (6-BA), naphthaleneacetic acid (NAA), and 2,4-dichlorophenoxyacetic acid (2,4-D), whereas the antibiotics used were carbenicillin, kanamycin and meropenem. The maximum callus induction rate was reached with less browning of leaf discs when a combination of the hormones 6-BA and 2,4-D at concentrations of 1 mg L^−1^ and 0.25 mg L^−1^, respectively, was used in the selection medium. This combination eventually resulted in the maximum regeneration of putative shoots. Increasing the concentration of 6-BA to 1.5–2 mg L^−1^ and 2,4-D to 0.3–0.4 mg L^−1^ resulted in a prominent decrease in the total number of shoots per explant and shoot regeneration percentage, as shown in Table 3. Furthermore, the effect of 6-BA and NAA combination on regeneration and genetic transformation percentage of the resistant shoots was examined; 0.8 mg L^−1^ NAA with 1 mg L^−1^ 6-BA was the most suitable combination for the maximum number of shoots, whereas a low concentration range of NAA 0.1–0.4 mg L^−1^ retarded the growth of the explants, as shown in Table 4. The comparison shows that the combination of 6-BA and 2,4-D generated a greater number of regenerated putative shoots than the combination of 6-BA and NAA.

**Figure 3:**
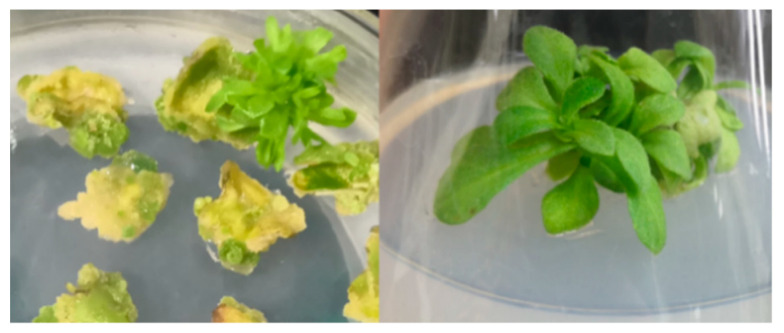
Developmental stages and screening of *CmTFL1a* transgenic plants of chrysanthemum cv. Jinba. Callus regeneration after 2 weeks on selection medium containing meropenem (50 mg L^−1^) and kanamycin (7 mg L^−1^). Induction of resistant adventitious shoots after 3 weeks. Kanamycin resistant shoots with large green leaves after 4 weeks.

**Figure 4:**
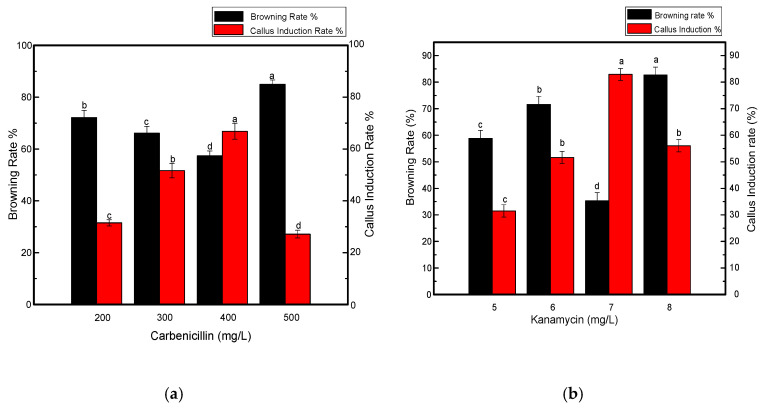

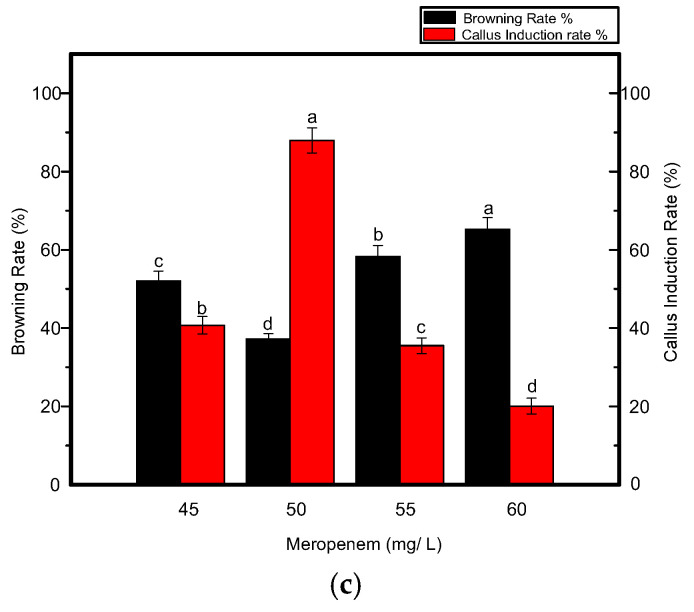
Effects of various antibiotics combinations on browning rate of leaf discs and callus induction from leaf discs of Chrysanthemum Jinba; (**a**) Effects of carbenicillin; (**b**) Effects of kanamycin; (**c**) Effects of meropenem. Black columns represent browning rate (%), red columns represent callus induction rate (%). Three biological replicates were used. Error bars represent ± SD. a, b, c, and d are statistically significant at *p* ≤ 0.05 by ANOVA on ranks test.

In addition to growth regulators, different selective agents were also tested at specific concentrations. Kanamycin at a concentration of 7 mg L^−1^ resulted in a significant increase in callus induction (Figure 3) when used along with carbenicillin at a concentration of 200 mg L^−1^, as shown in Figure 4a,b. Four out of 5 shoots were tested positive in each biological replicate. When the concentration of kanamycin was increased to 8–10 mg L^−1^, shoot regeneration was not significantly inhibited. However, more callus browning, followed by shoot development was prevalent. By further increasing the kanamycin concentration to 15 mg L^−1^, poor callus induction was observed and shoots could not regenerate even after 4–5 weeks. Eventually, necrosis was predominant when the kanamycin concentration was increased and varied between 15–25 mg L^−1^ in the medium. Another antibiotic, meropenem, was tested along with carbenicillin to investigate its effect on the effective removal of bacterial strains from transgenic calli. Although it is uncommonly used for shoot development, meropenem gave satisfactory results, especially when used at a minimal concentration of 50 mg L^−1^. A higher rate of callus induction was observed followed by a greater number of putative shoots (Figure 4c) with four shoots tested as positive per replication. Fewer regenerated calli and shoots were produced when the concentration of meropenem was increased to 55–60 mg L^−1^. Contrary to carbenicillin, regenerated shoots were not negatively affected by an increase in the meropenem concentration. From the above-mentioned results, it was determined that 7 mg L^−1^ kanamycin and 50 mg L^−1^ meropenem were appropriate concentrations for the effective selection of resistant transgenic Jinba plants.

### 3.4. Recognition of the Presence of Transgenes in Jinba Transgenic Plants

**Figure 5:**
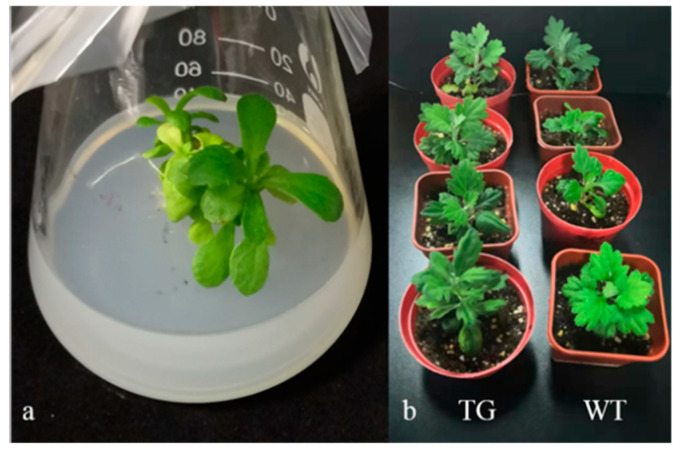
Positive *CmTFL1a* transgenic shoots of Chrysanthemum cultivar Jinba; (**a**) putative resistant shoot on rooting medium; (**b**) TG: Transgenic plants, WT: Wild type plants produced in a greenhouse.

**Figure 6:**
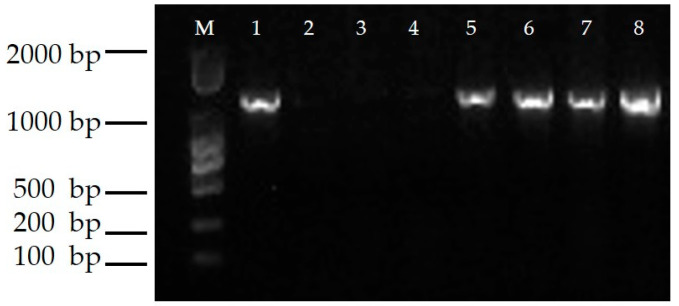
RT- PCR Analysis for the expression of *CsTFL1a* in transgenic lines, M: DL 2000 DNA Marker; 1: positive control, 2: WT, 3: double distilled (dd) H_2_O; 4–8: Transgenic resistant plants.

The presence of the transgene *CmTFL1a* was detected in transgenic shoots and, with PCR analysis, it was shown that all putative shoots contained fragments of 1100 bp in length. However, these fragments were absent in control plants. Positive *CmTFL1a* transgenic shoots are shown in Figure 5. Moreover, the outcomes of RT-PCR analysis exhibited constant expression of *CmTFL1a* in all transgenic lines #5, #6, #7 and #8, as shown in Figure 6.

### 3.5. Expression Pattern of CmTFL1a Gene and Comparison of Other Growth Trends in Transgenic Chrysanthemum Plants

**Figure 7:**
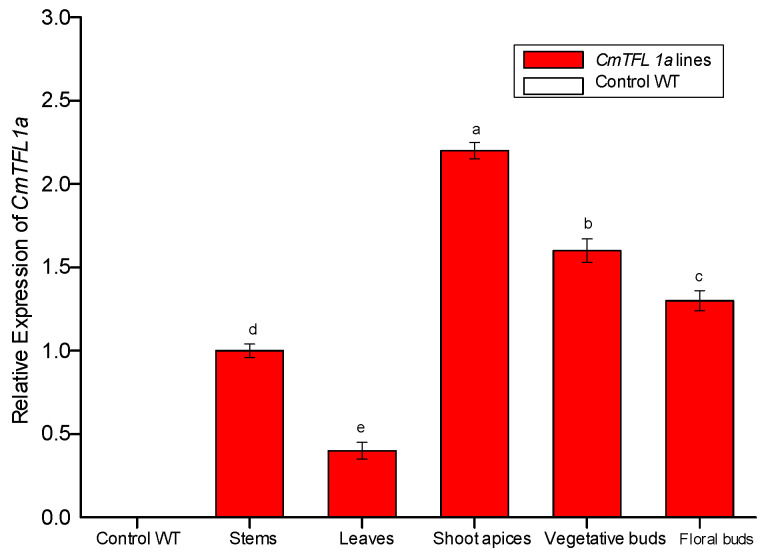
Relative expression of *CmTFL1a* in chrysanthemum cv. Jinba. Expression of *CmTFL1a* in various tissues of chrysanthemum by qPCR analysis. *Actin* was used as a reference gene. Three biological replicates were used for each sample. Error bars represent ± SD. a, b, c, d, and e are statistically significant at *p* ≤ 0.05 by ANOVA on ranks test.

**Figure 8:**
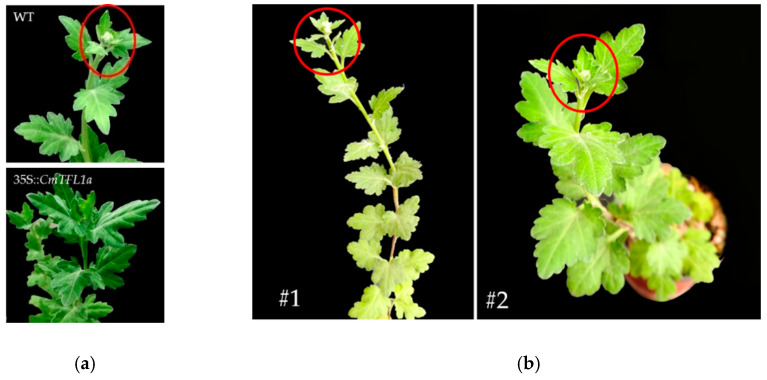

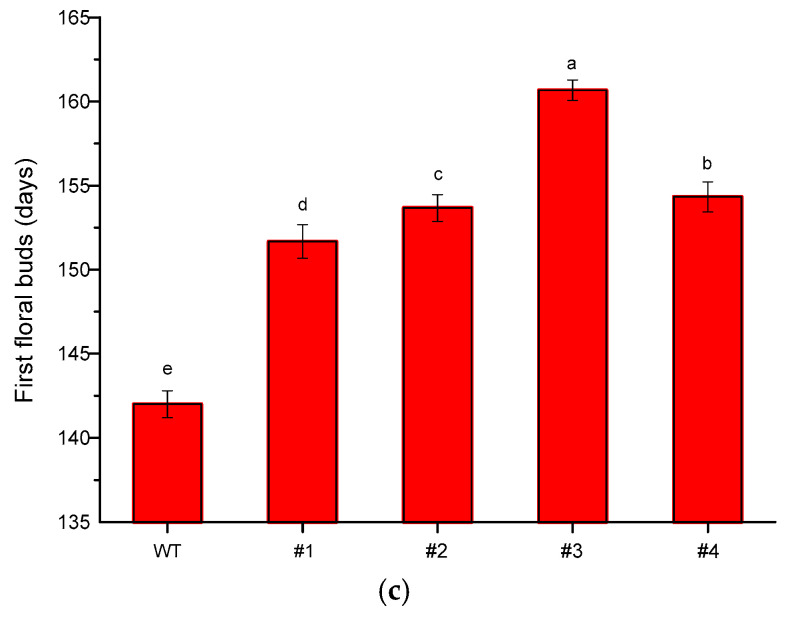
The flowering time of *CmTFL1a* transgenic chrysanthemum cultivar Jinba; (**a**) appearance of floral bud in wild chrysanthemum (upper) after 142 days in contrast with transgenic chrysanthemum (bottom); (**b**) floral bud formation after 151 and 153 days in transgenic lines #1 and #2, respectively; (**c**) floral budding period in wild chrysanthemum and in several transgenic lines. Three biological replicates were used. Error bars represent ± SD. a, b, c, d, and e are statistically significant at *p* ≤ 0.05 by ANOVA on ranks test.

qRT-PCR analysis was performed to examine the expression pattern of *CmTFL1a* in Jinba transgenic plants. After confirmation from PCR, a total of 4 transgenic lines of chrysanthemum (*CmTFL1a* #5, #6, #7, #8) were obtained and were selected to observe the relative expression level of the transgene comprehensively. The results show that *CmTFL1a* was absent in control WT plants, whereas it was highly expressed in shoot apices, followed by vegetative buds, floral buds and in the stems of *CmTFL1a* transgenic lines (Figure 7). The highest expression of *CmTFL1a* in shoot apices is related to the suppression of inflorescence development. Besides, phenotypic measurements were taken for wild-type and all *CmTFL1a* transgenic lines. Results on phenotyping depicted a rapid increase in the number of days to floral buds in transgenic lines, relative to WT plants. The floral buds in wild chrysanthemum appeared after 142 days, whereas no bud formation in *CmTFL1a* transgenic plants was seen at this stage, as shown in Figure 8a. The four transgenic lines produced floral buds in 151, 153, 160 and 154 days, about 10 to 20 days later than control plants (Figure 8b,c).

**Figure 9:**
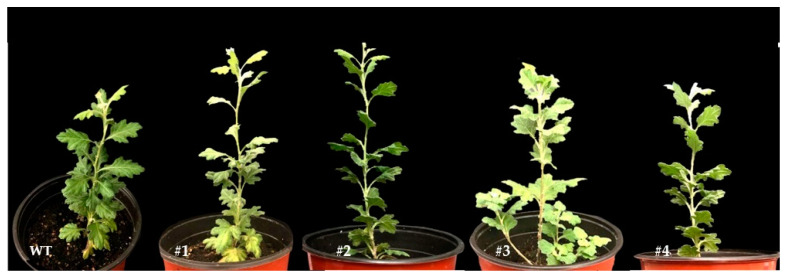
Phenotype of WT and *CmTFL1a* transgenic lines of Chrysanthemum. WT represents wild-type plant while #1-4 are positive *CmTFL1a* transgenic lines of Jinba.

**Figure 10:**
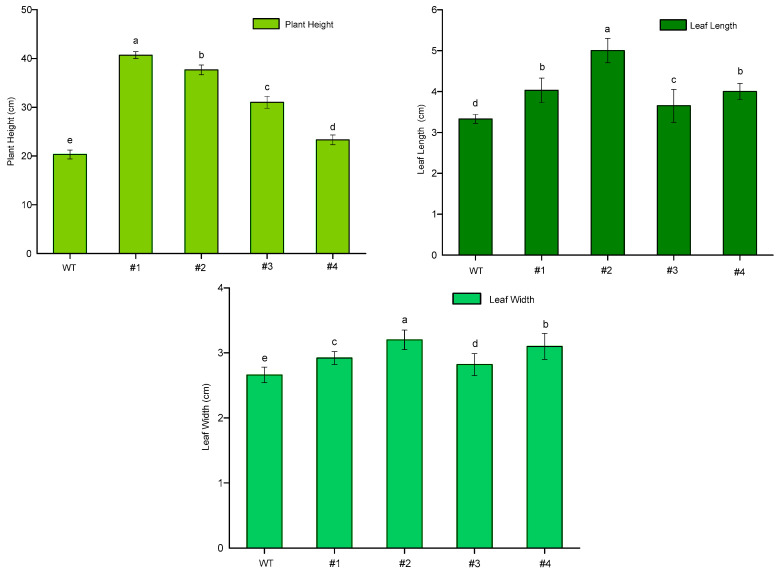
Growth potential (plant height, leaf length and leaf width in cm) of *CmTFL1a* transgenic chrysanthemum. Three biological replicates were used. Error bars represent ± SD. a, b, c, d, and e are statistically significant at *p* ≤ 0.05 by ANOVA on ranks test.

To observe and compare growth potential at the production of floral buds, plant height, leaf length and leaf width were recorded in both WT and transgenic plants. Consequently, plant heights and leaf length of *CmTFL1a* lines were significantly increased, in comparison with WT ones (Figure 9). The plant height of the WT plant was 22 cm, while the highest genetically modified chrysanthemum plant was 40 cm tall, as shown in (Figure 10). Among transgenic lines, strain #1 and #2 plants were significantly higher, while plant height differences between strain #3 and #4 were nonsignificant. When compared with WT plants, the leaf length and width of all transgenic lines were significantly different. These variations show higher growth potential of transgenic plants in comparison to the wild-type plants, which indicates that the trans-*CmTFL1a* gene can promote the growth of different tissue organs in chrysanthemum.

## 4. Discussion

Recently, unlike conventional breeding, the introduction of target genes for engineering crops has been made possible by genetic transformation techniques that do not require long-term periods of selection [32]. However, there have been few reports on crop improvement in chrysanthemum plants via the *Agrobacterium-*mediated transformation method [33,34,35].

### 4.1. Comparison between Standardized and Unstandardized Transformation Protocols

**Scheme 1:**
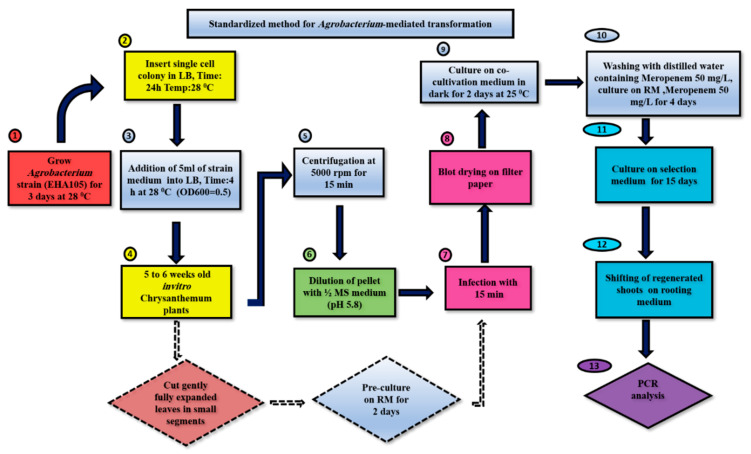
Standardized protocol for transformation in chrysanthemum cv. Jinba. Regeneration medium (RM): MS + 0.5 mg L^−1^ 6-BA + 0.5 mg L^−1^NAA, selection medium (SM): MS + 0.5 mg L^−1^ 6-BA + 0.5 mg L^−1^ NAA + 50 mg L^−1^ meropenem + 7 mg L^−1^ kanamycin, rooting medium: MS + 50 mg L^−1^ meropenem + 0.1 mg L^−1^ NAA.

In this study, we conducted a comparison between the unstandardized [29] and standardized protocols of *Agrobacterium*-mediated transformation for the chrysanthemum cultivar Jinba. Data for the optimal conditions of all individual parameters were recorded. The transformation of leaf explants of Jinba was performed by following both unstandardized and standardized methods. Different media compositions used in Naing et al. [29] were adapted and modified with new concentrations by following other literature, as mentioned in Section 2.3. The standardized protocol shown in Scheme 1 exhibited a higher regeneration and transformation percentage of shoots than the unstandardized protocol by using different selective agents at minimal concentration and generating a greater number of positive transgenic plants The salient parameters which were adapted and standardized in the current study were shoot regeneration, cocultivation, selection and rooting media composition, as well as cocultivation period and cocultivation temperature. Each of these parameters contributed to the extent of transformation efficiency in this cultivar. Above all, the standardized protocol resulted in a greater number of positive transgenic shoots in each replication, as confirmed by PCR analysis. Therefore, the standardization of individual features related to *Agrobacterium-*mediated transformation is equally imperative for improving the transformation efficiency in chrysanthemum cultivars.

### 4.2. Optimization of Parameters is Crucial for Higher Transformation Efficiency

Several studies have focused on the importance of cocultivation duration to improve the regeneration and transformation percentage of putative shoots [36], whereas it has been reported that the optimal cocultivation period is dependent on the explant genotype [37]. It was observed that coculturing of chrysanthemum (*Dendranthema grandiflora*) var. Micromargara explants for two days significantly affected their transformation efficiency [36]. A prolonged cocultivation period resulted in the overgrowth of bacteria. Thus, coculturing does not improve the transformation efficiency of plants [28].

The optimization of temperature conditions during cocultivation significantly affects transformation efficiency. The main reason is that the T-DNA transfer machinery works more efficiently at low temperatures. In this study, explants of Jinba exhibited the highest transformation efficiency when cocultured for 2 days at 22 °C. Our findings were consistent with some other reports that indicated that a temperature of 20 °C for a period of 2–3 days is optimal for orchid plant and *A. tumefaciens* cocultivation [38,39]. When the cocultivation temperature in this study was increased from 23 to 28 °C, the transformation efficiency was very low.

Few reports are available on the effects of various antibiotics on *Agrobacterium*-mediated transformation of Chrysanthemum. The exclusion of *Agrobacterium* from cultures is equally essential. The prolonged presence of *Agrobacterium* strains in cultured explants may decrease the cell proliferation rate resulting in the death of cells [30]. There are several antibiotics used to eliminate *Agrobacterium* cells from treated explants, for example, carbenicillin, cefotaxime, timentin and meropenem. Meropenem belongs to the carbapenem class of antibiotics that possesses extraordinary in vitro bactericidal activity against various microorganisms and is considered novel in removing *Agrobacterium* strains [40]. In this study, the authors examined the effects of two bactericidal antibiotics, i.e., meropenem and carbenicillin, on callus growth. It was found that a higher concentration of carbenicillin, 200 mg L^−1^, was required to control the regrowth of *Agrobacterium*. On the other hand, a much lower quantity of meropenem, 50 mg L^−1^, was required for the same purpose. It was confirmed that minor concentrations of meropenem are efficient in eliminating *Agrobacterium* cells from *Phalaenopsis* [30,40]. A higher concentration of carbenicillin may also lead to cell necrosis in the culture medium for two weeks. In contrast, meropenem effectively ceased bacterial growth at lower concentrations without any phytotoxicity.

For the selection of putative transgenic plants, as well as for improving shoot regeneration and transformation frequency of Jinba shoots, kanamycin was used at various concentrations, as the optimal concentration of kanamycin differs for each cultivar. It was reported that 30 mg L^−1^ kanamycin was optimal for the selection of *C. morifolium* var. Vivid Scarlet plants [14]. In addition, kanamycin prominently reduced shoot regeneration from stem segments of Yamabiko plants at a concentration of 10 mg L^−1^ [31]. In the current study, 7 mg L^−1^ kanamycin exhibited maximum callus induction followed by the greatest number of putative resistant shoots. However, 7.5–8 mg L^−1^ kanamycin completely inhibited callus induction and led to browning of treated explants.

### 4.3. Chrysanthemum CmTFL1a Delays Flowering and Regulate Growth Potential

*TFL1* controls the shoot apical meristem in the vegetative state of a plant [41]. During the juvenile period, it expresses in shoot apices that continue during floral bud and flower development [42]. The overexpression of the *CmTFL1* gene in chrysanthemum significantly delayed flowering [22,24], which shows that the *TFL1* gene has a conservative role in flowering regulation. Besides, the overexpression of the maize *ZEA CENTRORADIALIS* (ZCN) gene (belong to *the TFL1*-like family) extended the indeterminate growth of the inflorescence meristem, resulting in later floral transition [43]. In apple, the expression of *MdTFL1* and *MdTFL1a* was dominant in apical shoots during vegetative growth [44]. The *TFL1* gene was able to cause a significant delay in flowering and aided in the regulation of inflorescence architecture in several fruit tree species: citrus (*Citrus sinensis*) [45], apple (*Malus × domestica*) [46] and Japanese apricot *(Prunus mume*) [47]. Moreover, 35S:: *CmTFL1a* significantly improved the growth potential (plant height, leaf length, leaf width) of 35S:: *CmTFL1a* genetically modified plants as compared to wild plants. Specifically, an increase in plant height may be related to a high level of *CmTFL1a* gene expression in shoot apices, promoting the growth of the apical meristem. These results were consistent with the findings of [48].

## 5. Conclusions

In this work, the authors used an optimized method for regeneration and *Agrobacterium*-mediated transformation in the chrysanthemum cultivar Jinba. The optimized protocol can lead to a higher rate of transformation frequency as compared to an unstandardized one. Genetic transformation with *A. tumefaciens* by using leaf explants was effective in the construction of a standardized transformation scheme for Jinba. In summary, this study proposes that the evaluation of ideal conditions is crucial to increase the success rate of genetic transformation in chrysanthemum. Because of the lower concentrations and short period time for meropenem application, this method is the simplest as well as most cost-effective technique. In addition, maximum transgenic lines detected by RT-PCR showed stable expression of *CmTFL1a*. We assume that the protocol and conditions enhanced in this study will be helpful in the genetic engineering of this cultivar with various desirable genes. Furthermore, it will help to understand the biological phenomenon of the *TFL1* homolog in chrysanthemum.

## Figures and Tables

**Table 1:** Various concentrations of hormones and antibiotics used in MS medium for chrysanthemum transformation.

Hormones concentrations (mg L^−1^)		Antibiotics Concentrations (mg L^−1^)			
6-BA	2,4-D	NAA	Car	Mer	Ka
* **0.5**	0.1	* **0.1**	* **200**	45	6
1	* **0.25**	0.2	300	* **50**	* **7**
1.5	0.4	0.5	400	55	8

* These concentrations were used as controls; 6-BA: 6-benzylaminopurine; 2,4-D: 2,4-dichlorophenoxyacetic acid; NAA: naphthaleneacetic acid; Car: carbenicillin; Mer: meropenem; Ka: kanamycin.

**Table 2:** Primers and polymerase chain reaction (PCR) parameters used for the current study.

Genes	Primers	PCR Conditions
*CmTFL1a*	*CmTFL1a*-F CCA ACA ACC CAA CTT GTT AAA A	98 °C for 30 s, followed by undergoing 25 cycles of 98 °C for 10 s, 60 °C for 30 s
	*CmTFL1a*-R CGA CAG AAT TTG ACC AAA AG	
*CmTFL1a-Q*	*CmTFL1a*-QF CCT CGT GTT GAT ATC GGT	98 °C for 30 s, followed by undergoing 25 cycles of 98 °C for 10 s, 60 °C for 30 s
	*CmTFL1a*-QR GTA ACA ATC CAG TGA AGA TGT	
*Actin*	*Actin*-F GGT GTC ATG GTT GGT ATG GGTC	95 °C for 2 min, followed by 30 cycles of 95 °C for 20 s, 57 °C for 40 s, and 72 °C for 1 min
*CmTFL1a-pmi*	*Actin*-R CCT CTG TGA GTA GAA CTG GGTGC *CmTFL1a-pmi*-F GAT GTT TAG ATA GAG ACT CGA GAT GGC AAG ATT AAC ATT AAC TTC GGG TCA	95 °C for 2 min, followed by 30 cycles of 95 °C for 20 s, 57 °C for 40 s, and 72 °C for 1 min
	*CmTFL1a-pmi*-R AGT GTG TAA TAC CTC TTT CTC CAG TTA TCG TCT GGG AGC TGT TTC TCT T	

**Table 3:** Role of 6-benzylaminopurine (6-BA) and 2,4-D hormones combinations in browning rate of leaves, callus induction rate and the average number of regenerated shoots of Jinba.

Growth Regulators		Browning Rate of Leaves (%)	Callus Induction Rate (%)	Average Number of Regenerated Shoots
6-BA	2,4-D			
0.5	0.1	71.33 ab^x^	0.00 e	0
0.5	0.25	78.60 a	1.50 de	0
0.5	0.4	66.43 bc	4.1 de	1 f
1	0.1	14.96 f	42.4 b	1.77 e
1	0.25	4.32 g	61.43 a	1.96 a
1	0.4	31.7 e	46.03 b	1.84 b
1.5	0.1	49.53 d	31.83 c	1.62 c
1.5	0.25	62.46 c	5.76 d	1.24 d
1.5	0.4	72.63 ab	0.33 e	0

^x^ Mean totals with different letters are different (*p* ≤ 0.05) by ANOVA on ranks test.

**Table 4:** Effects of 6-BA and NAA hormones combinations on browning rate of leaves, callus induction rate, and the average number of regenerated shoots of Jinba.

Growth Regulators		Browning Rate of Leaves (%)	Callus Induction Rate (%)	Average Number of Regenerated Shoots
6-BA	NAA			
0.5	0.1	66.46 e^x^	7.6 cd	1.00 f
0.5	0.4	85.47 b	28.6 b	1.62 b
0.5	0.8	71.43 d	10.53 c	1.13 e
1	0.1	82.8 c	0.00 d	0
1	0.4	72.0 d	10.82 c	1.22 cd
1	0.8	22.93 f	50.73 a	1.75 a
1.5	0.1	70.90 d	13.5 c	1.3 d
1.5	0.4	91.4 a	0.00 d	0
1.5	0.8	87.1 b	13.73 c	1.48 c

^x^ Mean totals with different letters are different (*p* ≤ 0.05) by ANOVA on ranks test.
